# Colocolic intussusception due to lipoma in an adolescent with refractory anal fissure; case report and literature review

**DOI:** 10.1016/j.ijscr.2024.109759

**Published:** 2024-05-14

**Authors:** Behzad Nematihonar, Leily Mohajerzadeh, Tahmaseb Jouzdani, Hojatolah Khoshnoudi, Seyed Pedram Kouchak Hosseini, Alireza Haghbin Toutounchi

**Affiliations:** a Department of General Surgery, Imam Hosein Medical and Educational Center, Shahid Beheshti University of Medical Sciences, Tehran, Iran; b Pediatric Surgery Research Center, Research Institute for Children's Health, Shahid Beheshti University of Medical Sciences, Tehran, Iran

**Keywords:** Intussusception, Invagination, Constipation, Intestinal lipoma, Anal fissure

## Abstract

**Introduction and importance:**

Intussusception is uncommon in older patients, making its diagnosis challenging and necessitating a high level of clinical suspicion. While pediatric intussusception typically presents with a triad of symptoms including abdominal pain, bloody diarrhea, and an abdominal mass, the majority of adult patients experience chronic abdominal pain and partial obstruction. Consequently, the diagnosis of adult intussusception may be delayed due to the similarity in presentation with other conditions.

**Case presentation:**

In this article, we have presented a 13-year-old boy with chronic and refractory anal fissure. The patients also complained of constipation for a year, intermittent abdominal pain, and bloating. Although he was treated with conservative laxative medications, the constipation was not relieved. Incidentally, a colocolic intussusception was found through an MRI.

**Clinical discussion:**

We have provided a comprehensive description of an unexpected intussusception at an uncommon age which was found incidental. Medical literature was reviewed for better optimal planning in surgery.

**Conclusion:**

Intussusception in a teenager is unexpected, and this case shows the importance of considering it even in the presence of nonspecific symptoms. This case serves as a reminder to healthcare professionals to consider intussusception as a potential diagnosis in similar cases.

## Highlights

• Consequently, the diagnosis of adult intussusception may be delayed due to the similarity in presentation with other conditions.
• Lipomas, generally asymptomatic, but they can occasionally lead to complications such as intussusception.
• Intussusception in an adolescent is less expected, and this case shows the importance of considering it even in the presence of nonspecific symptoms.
• This case serves as a reminder to healthcare professionals to consider intussusception as a potential diagnosis in similar cases.

## 1 Introduction

Intussusception occurs when a segment of the intestine (intussusceptum), typically with a mesenteric fold, telescopes into the lumen of an adjacent portion of the intestine (intussuscipiens) due to peristalsis [1]. While more commonly seen in pediatrics, it can still occur rarely in adults, with an estimated incidence of 2–3 cases per 1,000,000 population per year [2]. In adults, the clinical presentation differs from that in pediatrics, often lacking the classic triad of abdominal pain, palpable abdominal mass, and hematochezia, which complicates diagnosis [1].

While intussusception tends to be benign in pediatric cases, underlying pathology is identified in up to 90 % of adult cases [3]. Benign tumors, such as lipomas, are less common, with the colon being the most frequent site. Lipomas, generally asymptomatic, are often incidentally discovered during colonoscopy, surgery, or autopsy. However, they can occasionally lead to complications such as abdominal pain, diarrhea, constipation, mimicking colon cancer, and even causing intussusception [1]. We have reported an unexpected case of intussusception in a 13-year-old boy. The work has been reported in line with the SCARE 2023 criteria [4].

## 2 Case presentation

### 2.1 History

A 13-year-old boy presented in the clinic of surgery with chronic and refractory anal fissure. The patients also complained of constipation for a year, intermittent abdominal pain, and bloating. He had no medical or surgical history, and his parents did not mention any remarkable medical issues at birth, hospitalization, or any problems in case of digestion, or defecation before. No history of nausea, vomiting, loss of appetite, or losing weight. Although he was treated conservatively with laxative medications in the past 3 months, the constipation was not relieved.

### 2.2 Assessment

**Fig. 1:**
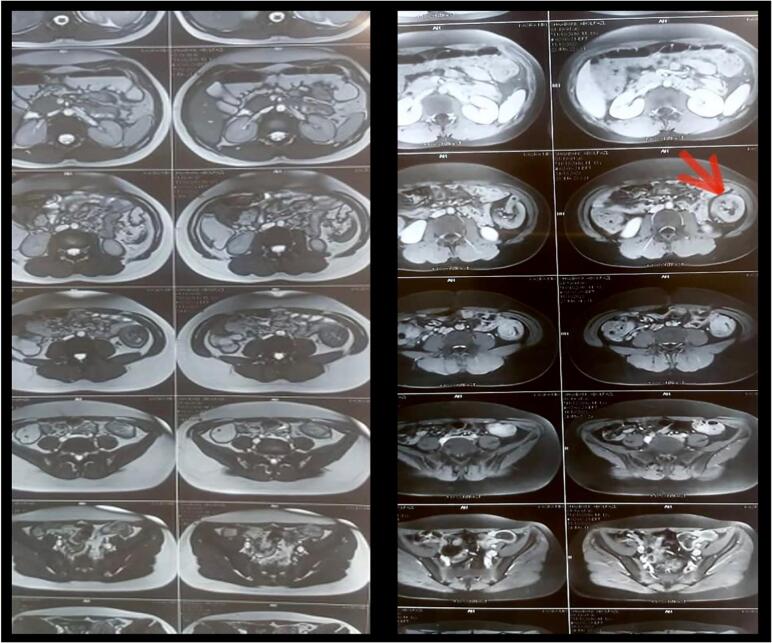
MR Enterography shows the view of the target sign at the site of descending colon which is specific for intussusception.

**Fig. 2:**
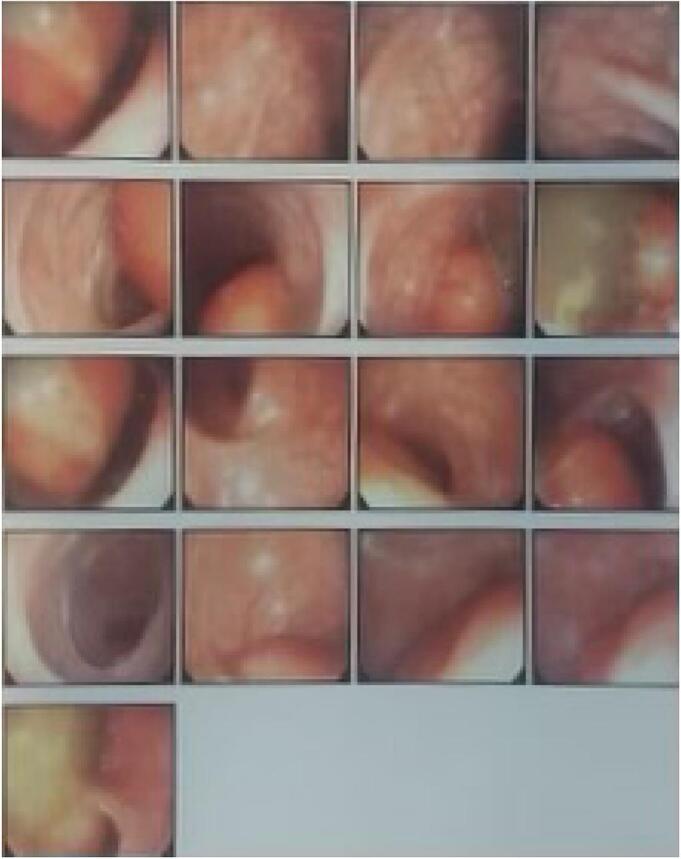
Colonoscopy revealed intussusception and a suspicious mass.

According to the history of chronic constipation and refractory anal fissure, an MR Enterography was performed for surveying inflammatory bowel diseases. All small bowel loops including duodenum, jejunum, and ileum had normal appearance and distribution with normal wall thickness and mucosal pattern. There was no evidence of wall thickening or inflammatory changes. Multiple small lymph nodes were evident in ileocecal mesentery the largest one has 16*10 mm diameter. The MRI remarked an unexpected mass-like lesion in descending and sigmoid colon with a fat component that was suspicious of intussusception (Fig. 1). A total colonoscopy was performed to confirm the intussusception and to find the probable cause of the lead point. The colonoscopy resulted in a longitudinal mass-like lesion in sigmoid colon with length of 12 cm and intussusception at the site of sigmoid and descending colon (Fig. 2). Finally, the patient was prepared for laparoscopy surgery after pediatric surgeon consult with diagnosis of colocolic intussusception due to an intraluminal lesion.

### 2.3 Operation summary

**Fig. 3:**
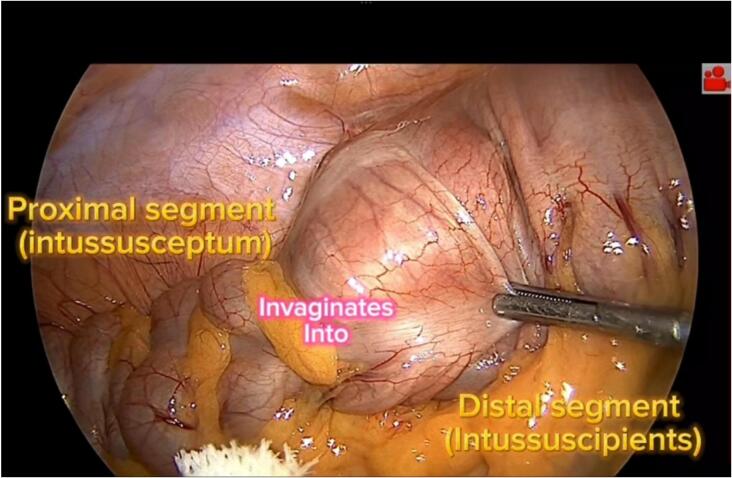
View of the invagination site through the laparoscope.

**Fig. 4:**
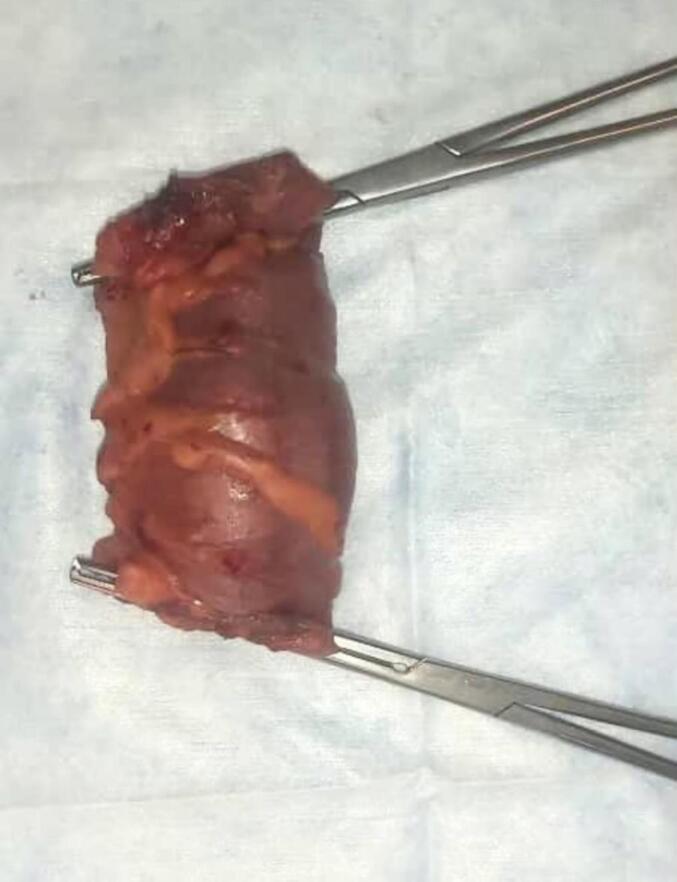
Resected segment of colon including the lipoma.

Laparoscopic surgery was performed under general anesthesia in line with pediatric protocols and standard trocars. The peritoneal cavity was explored, intussusception site and lead point were detected (Fig. 3). While the invagination was chronic and to illuminate the suspect lesion and take lymph nodes at the same time for Pathology, a segmental resection (Fig. 4) and anastomosis were performed by the hybrid hand-assisted surgery through the wound retractor (Video 1).

### 2.4 Outcome

The pathology reported only a submucosal lipoma and 8 reactive lymph nodes without the presence of malignancy or inflammatory changes. The patients were discharged in a few days without complications. At3 months later follow-up, constipation was relieved, and the anal fissure improved.

## 3 Discussion

Adult intussusception is indeed a rare occurrence, with an estimated incidence of 2–3 cases per 1,000,000 population annually [1]. Unlike pediatric cases, which are often primary or idiopathic, adult intussusception is predominantly attributed to structural lesions acting as lead points [1]. Among adults, malignant neoplasms serve as the primary lead points, with adenocarcinoma being the most common cause in colonic intussusception, while metastatic masses are more frequently encountered in the small bowel [5]. Other less common causes include benign tumors such as lipomas, adenomatous polyps, fibromas, leiomyomas, and hamartomas, as well as conditions like post-surgical adhesions, lymphoid hyperplasia, cystic fibrosis, scleroderma, celiac disease, inflammatory bowel disease (e.g., Crohn's disease), appendicitis, pancreatitis, and rectal foreign bodies [2]. In the majority of adult intussusception cases, the development is associated with a focal area of traction, often due to a polyp or cancer, which pulls the proximal bowel into the distal bowel during peristalsis, leading to bowel ischemia and subsequent complications such as bowel necrosis and sepsis [2]. In the presented case, a large lipoma was identified as the underlying cause.

Lipomas, which are non-epithelial benign masses comprised of adipose cells, can be found throughout the gastrointestinal tract, with the colon being the most common site [1,2]. Intestinal lipomas are rare, with reported incidences ranging from 0.2 % to 4.4 %, and although they can occur in the colon, they are an infrequent cause of intussusception [5]. Typically, these neoplasms appear as sessile polypoid masses, occasionally pedunculated, with sizes varying from 2 mm to 30 cm [1]. Symptoms usually arise when the lipoma is>2 cm in diameter, potentially causing intestinal obstruction, bleeding, or intussusception [2,5]. Previous research indicates that colonic lipomas most commonly manifest in the ascending colon (45 %), followed by the sigmoid colon (30.3 %) and descending colon (15.2 %) [2]. Lipomas of the transverse colon, such as in the case of our patient, are the least common, accounting for only 9.1 % of occurrences [2].

Intussusception is classified into four subtypes based on the involved bowel segments: enteric, ileocolic, colocolonic, and sigmoidorectal intussusception [3]. Colocolonic intussusception, though less common than small bowel intussusception [5]. However, it is more likely to be associated with a malignant lead point compared to small bowel intussusception, according to the higher prevalence of malignancies in the colon. Ileocolic intussusception in adults represents a distinct variant, with almost all cases (nearly 100 %) being associated with a malignant lead point [5].

Intussusception is uncommon in older patients, making its diagnosis challenging and necessitating a high level of clinical suspicion. While pediatric intussusception typically presents with a classic triad of symptoms including abdominal pain, bloody diarrhea, and a palpable mass, the majority of adult patients experience chronic abdominal pain and partial obstruction [3]. <20 % of adult intussusception cases result in acute complete bowel obstruction, and around 40 % exhibit a palpable abdominal mass [2]. Adult intussusception often presents as chronic intermittent abdominal pain accompanied by nonspecific signs of bowel obstruction, as seen in the current patient [3]. Given the nonspecific symptoms and signs associated with intussusception, differential diagnoses such as inflammatory bowel disease (IBD), appendicitis, and volvulus should also be considered [3]. Consequently, the diagnosis of adult intussusception may be delayed due to the similarity in presentation with other conditions.

Ultrasound serves as the initial imaging modality for pediatric patients due to its advantages, including rapid and cost-effective real-time examination. However, its efficacy can be influenced by operator proficiency and bowel distention. Classic ultrasound features of intussusception include the target or doughnut sign, crescent in a donut sign, and pseudo-kidney sign. Nonetheless, ultrasound tends to be less accurate in adults, especially in cases of massive air or morbid obesity [5]. In adults, the evaluation typically begins with an abdominal radiography, which may reveal signs of intestinal obstruction or perforation, providing information on the obstruction's location. CT scans often demonstrate characteristic findings such as target, bulls-eye, or sausage-shaped lesions, indicative of the bowel-within-bowel appearance caused by the anatomic arrangement of the outer intussuscipiens and central intussusceptum. A CT scan can also reveal impaired perfusion, venous stasis, edema, and bowel wall air associated with necrosis or gangrene. Additionally, CT scan can differentiate intussusception with a lead point, such as a tumor, although it has limited ability to remark the nature of the lesion (malignant, benign, or idiopathic). Despite advances in medical imaging, discriminating between malignant tumors and large lipomas preoperatively remains challenging, and histopathology is often required for definitive diagnosis [1]. Exploratory laparotomy typically precedes diagnosis, as only about 50 % of cases are diagnosed preoperatively [2].

In pediatrics, nonoperative reduction via air or contrast enemas is the typical management approach for intussusception [2]. Additionally, colonoscopy is utilized as both a diagnostic and therapeutic modality. Treatment for pediatric intussusception often leans towards conservative management, with reduction achieved through barium enemas rather than resorting to surgical intervention. In contrast, due to the higher incidence of lead point involvement and underlying malignancy in adults, surgical intervention is typically required for the treatment of intussusception. The specific approach varies depending on the lesion's location. While enteric intussusception often necessitates surgery, the management of colocolonic intussusception is subject to debate [3]. Given the frequent presence of underlying pathology, laparotomy is often preferred over reduction. However, there is some contention regarding whether en-bloc resection or lesion reduction should be prioritized, with concerns about the risk of transperitoneal, vascular, and intraluminal seeding. Nevertheless, proponents of lesion reduction argue that it may reduce the need for extensive bowel resection [2]. Regardless of the subtype of intussusception, surgery is needed when enema reduction, or when imaging studies reveal signs of bowel necrosis, peritonitis, or the presence of a lead point mass [5].

## 4 Conclusion

Intussusception in a teenager is less expected, and this case shows the importance of considering it even in the presence of nonspecific symptoms. The patient's condition was initially missed, highlighting the challenge of arriving at the correct diagnosis. It was only incidentally discovered while investigating other problems. This case serves as a reminder to healthcare professionals to consider intussusception as a potential diagnosis in similar cases, even when symptoms are not typical. Such awareness can lead to timely recognition and appropriate management, ultimately improving patient outcomes.

The following are the supplementary data related to this article.

Video 1 Laparoscopic surgery was performed under general anesthesia in line with pediatric protocols and standard trocars. The peritoneal cavity was explored, intussusception site and lead point were detected. A segmental resection and anastomosis were performed by the hybrid hand-assisted surgery through the wound retractor. Video 1

## Provenance and peer review

Not commissioned, externally peer reviewed.

## Patient consent

Written informed consent was obtained from the patient for publication of this case report and accompanying images. A copy of the written consent is available for review by the Editor-in-Chief of this journal on request.

## Ethical approval

Ethical approval is exempt/waived at our institution (Shahid Beheshti University of Medical Sciences, Iran) for de-identified case reports.

## Funding

This article did not receive funds.

## Guarantor

Dr. Behzad Nematihonar accepts all responsibility of this article.

## Research registration number

N/A.

## CRediT authorship contribution statement

Dr. Behzad Nematihonar, participated in supervision and validation.

Dr. Leily Mohajerzadeh, participated in conceptualization and methodology.

Dr. Tahmaseb Jouzdani, participated in writing - original draft and writing - review & editing.

Dr. Hojatolah Khoshnoudi, participated in visualization and writing - review & editing.

Dr. Seyed Pedram Kouchak Hosseini, participated in term and data curation.

Dr. Alireza Haghbin Toutounchi, participated in writing - original draft, project administration.

## Declaration of competing interest

All authors declare that they have no conflicts of interest.
